# Serum Anti-Müllerian Hormone Levels in Patients with Epithelial Ovarian Cancer

**DOI:** 10.1155/2013/517239

**Published:** 2013-07-28

**Authors:** Pawel Walentowicz, Pawel Sadlecki, Magdalena Krintus, Grazyna Sypniewska, Aneta Mankowska-Cyl, Marek Grabiec, Malgorzata Walentowicz-Sadlecka

**Affiliations:** ^1^Department of Obstetrics and Gynecology, L. Rydygier Collegium Medicum in Bydgoszcz, Nicolaus Copernicus University, Ulica Ujejskiego 75, 85-168 Bydgoszcz, Poland; ^2^Department of Laboratory Medicine, L. Rydygier Collegium Medicum in Bydgoszcz, Nicolaus Copernicus University, Ulica Jurasza 5, 85-094 Bydgoszcz, Poland

## Abstract

*Objectives*. The aim of our study was to examine serum anti-Müllerian hormone (AMH) concentration in ovarian cancer patients in relation to clinicopathological features, such as a pathological subtype of the tumor, (FIGO) stage, grading, and overall 5-year survival. *Material and Methods*. We enrolled 72 epithelial ovarian cancer patients in our study, aged 45–79 years, who underwent optimal cytoreductive surgery. In all patients, serum AMH concentration was measured using a two-step sandwich type enzyme immunoassay before surgery. As a reference value for women over 45 years we accepted anti-Müllerian hormone concentration below 1 ng/mL. *Results*. In the whole group of patients with ovarian cancer, median serum concentration of AMH was 0.07 (0.0–0.37) ng/mL, whereas in the group of those with positive AMH values (≥0.14 ng/mL) it was 0.31 (0.15–0.73) ng/mL. No significant correlation was found between serum AMH levels and FIGO stage, histological subtype, or grading (*P* > 0.05). The analysis of five-year survival rate related to AMH levels showed no statistically significant differences. There were no differences in survival rates between patients with positive or negative serum AMH levels. *Conclusion.* Measurement of serum anti-Müllerian hormone levels was not useful in predicting clinicopathological features and survival in patients with ovarian cancer.

## 1. Introduction

Among cancers of female reproductive system, ovarian cancer became the second most common and is the fifth ranked cause of cancer-related mortalities in women in Europe and the United States [1]. Although understanding of ovarian cancer has improved substantially, the etiology of the disease remains unknown. Moreover, even though surgical procedures made a great progress and new protocols of chemotherapy were introduced, the 5-year survival rate does not exceed 45%. The main reason for this situation is that the majority of women with ovarian cancer turn to gynecologist for help at the late stage of the disease and that despite introducing new markers for the detection of ovarian cancer, their low diagnostic sensitivity does not permit to use them as screening [2, 3].

Anti-Müllerian hormone (AMH), also known as Müllerian inhibiting substance (MIS), belongs to a larger family of transforming growth factor-*β* (TGF-*β*). AMH signals through two transmembrane receptors, type II which is specific (present in Müllerian duct and gonads) and type I receptors, shared with the bone morphogenetic proteins family [4, 5].

AMH is expressed in the Sertoli cells of fetal testis from the seventh week of pregnancy [6], and its secretion is fundamental in regression of the Müllerian ducts [7].

In women AMH is produced by granulosa cells, from preantral and antral follicles. Serum AMH levels are undetectable in newborns, increase during childhood and adolescence reaching its peak in the early 20s, and remain stable throughout reproductive period only to decrease during menopausal transition [8, 9]. During menstrual cycle serum AMH maintains its level and lowers slightly in early secretory phase [10, 11].

Studies on mice had shown that AMH inhibits the transition from the primordial to the primary follicular stage. AMH paracrine signaling inhibits FSH-related follicle growth leading to selection of primary follicle. FSH and estradiol decrease AMH gene expression in granulosa cells [12]. Recent in vitro studies confirmed that increased expression of AMH is caused by bone morphogenetic protein (BMP-6) and also that AMH inhibits recruitment of primordial follicles in order to keep ovarian function in balance [13].

The number of primordial follicles decreases with age of women. Lower serum AMH levels precede the increase of FSH and inhibin B levels and thus are the most accurate parameter of ovarian reserve in clinical practice [14–16]. AMH possesses high prognostic value in prediction of number of obtained oocytes [17, 18]. Besides, ovarian hyperstimulation syndrome seems to be associated with significantly higher basal AMH levels [19].

In women undergoing oncologic treatment AMH is considered a useful marker of damage to the ovarian reserve [20, 21].

The fact that AMH expression is restricted to ovarian granulosa cells in women led to establishing AMH levels as a serum marker of granulosa cell tumours (GTCs). Recent studies showed an increased serum AMH concentration in 76–93% of women with both primary and recurrent GTCs [22, 23].

As anti-Müllerian hormone belongs to transforming growth factor-*β* family and its representatives play an important role in ovarian cancer carcinogenesis, the aim of this study was to examine AMH concentration in ovarian cancer patients in relation to clinicopathological features, such as a pathological subtype of the tumor, FIGO stage, grading, and overall 5-year survival.

## 2. Patients, Materials, and Methods

### 2.1. Patients

We enrolled 72 epithelial ovarian cancer patients aged 45–79 years (mean 58.5 ± 10 years) treated in Department of Gynecology and Obstetrics, Ludwik Rydygier Collegium Medicum in Bydgoszcz in the period of 2005-2006. 

Only women who underwent optimal cytoreductive surgery were considered for further analysis, all of them with residual cancerous focuses smaller than 1 cm in diameter. All patients were operated by experienced gynecological oncologist. The standard surgical protocol included tumorectomy, hysterectomy with bilateral salpingoovariectomy, pelvic lymphadenectomy, omentectomy, and appendectomy. 

Women who had ever used hormonal replacement therapy or had undergone ovulation induction were excluded. All patients were Caucasian, after menopause with BMI range from 19 to 30 kg/m².

International Federation of Gynecology and Obstetrics (FIGO) ovarian cancer staging system was used to assess clinical stage of the disease. Early stages were confirmed in 16 patients (FIGO I in 9 women, II in 7) and advanced disease in 56 patients (FIGO III—50, IV—6). All ovarian cancer cases were with histological confirmation, of which 59 (82%) serous, 5 (7%) mucinous, and 8 (11%) endometrioid. Histological examination was performed in the Department of Pathology, Antoni Jurasz University Hospital in Bydgoszcz, and histological grading was determined (G1 in 9, G2 in 23, and G3 in 40 patients). 

After optimal cytoreductive surgery, all women underwent 6 courses of chemotherapy based on carboplatin and paclitaxel. Baseline characteristics of the study participants are shown in Table 1.

The Bioethical Committee at the Ludwik Rydygier Collegium Medicum, Nicolaus Copernicus University of Torun, have reviewed and approved this study. All participants have provided informed consent.

### 2.2. Methods

Blood samples were collected (10 mL) after admission to the hospital, on the day before surgery. After centrifugation in standard conditions serum was obtained, aliquoted, and stored at −70°C until assayed.

Serum AMH concentration was measured using a two-step sandwich type enzyme immunoassay (Immunotech AMH/MIS ELISA kit, Beckman Coulter). The imprecision of the assay was 12.3% at 0.2 ng/mL and 5.1% at 15.8 ng/mL. The lowest AMH concentration in a sample which could be detected with a 95% probability was 0.08 ng/mL (lower detection limit). Concentration of AMH below 0.14 ng/mL has been accepted as negative. 

Reference values for anti-Müllerian hormone for women over 45 years were less than 1 ng/mL [24].

The Kolmogorov-Smirnov test was used to assess normality of distribution of investigated parameters. Data were expressed as mean ± standard deviation and median with 25th–75th percentiles. Comparison between the groups was performed by using the Mann-Whitney U test and the Kruskal-Wallis test for non-Gaussian distributed variables. *P* value <0.05 was considered statistically significant. 

Overall survival rate was examined for significance using log-rank test and Kaplan-Meier curves. 

Univariate and multivariate Cox regressions were performed. For the analysis, a forward selection with a *P* value of less than 0.05 for entry was applied. The effects of the variables were expressed as hazard ratios per 1 SD change to allow for a better comparability between the effect sizes of the different tested variables.

All statistical analyses were performed using Statistica for Windows Statsoft 10.0. 

## 3. Results

In the group of patients with ovarian cancer median serum concentration of AMH was 0.07 (0.0–0.37) ng/mL, whereas median concentration in the patients with positive AMH values (≥0.14 ng/mL) was 0.31 (0.15–0.73). Values equal to/above 0.14 ng/mL were found in 44 women (61%).

Median concentrations of serum AMH in relation to FIGO stage did not differ significantly (Table 2).

No significant correlations were found between serum AMH concentration and histopathological subtype or grading (Tables 3 and 4).

The results of Cox regression of the predictive power of variables are shown in Tables 5 and 6. In the univariate analysis grading and FIGO were significantly correlated with survival time in women with ovarian cancer. In contrast, in the multivariate analysis only FIGO stage had a statistically significant effect on survival time. 

**Figure 1:**
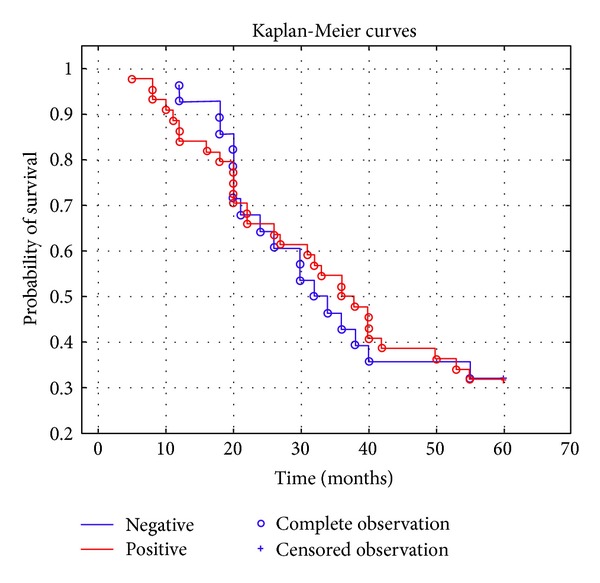
Survival curves in relation to AMH levels.

Overall survival rate was also examined in relation to AMH level. Kaplan-Meier analysis was performed in two groups of ovarian cancer patients: first group (*N* = 28) of women with serum concentration below the detection limit and another (*N* = 44) who displayed values over 0.14 ng/mL (Figure 1). The long-rank test showed no statistical significance (*P* = 0.98). The analysis of five-year survival rate related to AMH levels showed no statistically significant differences; there were no differences in survival rates between patients with positive or negative AMH values (Figure 1).

## 4. Discussion

 Although it was commonly believed that the cells of epithelial ovarian cancer come from the epithelium covering the surface of the ovary, the current theory states that cancer arises from the cells of the Fallopian tube. Most recent studies indicate that the vast majority of ovarian tumors derive from the fimbriae of Fallopian tube and other components developed from the Müllerian ducts [25]. Anti-Müllerian hormone (AMH) initiates the process of regression of Müllerian ducts. Based on these facts some authors suggest that the determination of AMH could be important in the diagnosis and treatment of epithelial ovarian cancer [26, 27]. 

The median concentration of AMH in epithelial ovarian cancer patients was 0.07 ng/mL taking into account both positive and negative AMH results. However, in the group of women with AMH levels above 0.14 ng/mL, the median was 0.31 (0.15–0.73). The results observed in our study were similar to those of age-specific AMH values found by others but in healthy women [24]. 

Despite the lack of statistically significant differences, women with FIGO classifications I and II had lower AMH levels than women with FIGO stages III and IV. On the other hand, according to histological grading, highest AMH values were observed in women with G1 staging.

 Detailed analysis of concentrations of anti-Müllerian hormone has revealed no differences in the levels of AMH, depending on the type of cancer, clinical stage, and histological grade. There was no correlation between serum AMH and the five-year survival rate. There were no differences in years of survival of patients with AMH in serum compared to patients who were negative for anti-Müllerian hormone. In our study only advanced clinical stage according to FIGO was an independent poor prognostic factor.

 To the best of our knowledge there are no available studies demonstrating the usefulness of determination of AMH concentrations in the serum of patients with epithelial ovarian cancer.

 The search for alternative therapies in the treatment of ovarian cancer has led to research on biologically active substances which might inhibit the proliferation of the tumor. Masiakos et al. hypothesized that the anti-Müllerian hormone may serve as such factor because it causes apoptosis and the regression of the Müllerian ducts in embryos, by variation of ovarian tumor cell biology [26]. Further studies showed the presence of type II receptor for AMH in the ovary and in ovarian cancer cells, and its inhibitory effect was confirmed in transgenic mice. In addition, tests were performed on cells obtained from peritoneal fluid from patients with ovarian cancer at FIGO stages III/IV. Masiakos et al. are of the opinion that the determination of AMH and its receptor by flow cytometry could be used in selection of patients with poor prognosis, but this fact has not been confirmed in subsequent studies [26].

 In conclusion, AMH belongs to the family of transforming growth factor, which plays an important role in ovarian carcinogenesis. However, none of the limited studies could demonstrate the role of the anti-Müllerian hormone in the serum of patients with ovarian cancer. Similarly, in our study, we failed to show any benefits from the determination of serum AMH in women with ovarian cancer.

Despite our findings concerning epithelial ovarian cancer, serum AMH levels remain well-established marker in granulosa-theca cell tumors. It is necessary to perform further studies to determine the tissue expression of AMH and type II receptor for AMH in ovarian cancer tissue and the potential usefulness of monoclonal antibodies against AMH and its receptor in the diagnosis and treatment of women with ovarian cancer.

## Figures and Tables

**Table 1:** Baseline characteristics of the study group.

Parameter	Patients with ovarian cancer *n* = 72
Age (years)	58.5 ± 10.4
BMI (kg/m^2^)	22 ± 2.2
FIGO stage *n* (%)	
I	9 (12.5%)
II	7 (10%)
III	50 (69.5%)
IV	6 (8%)
Histological subtype *n* (%)	
Serosum	59 (82%)
Mucinous	5 (7%)
Endometrioid	8 (11%)
Histological grading *n* (%)	
G1	9 (12.5%)
G2	23 (32%)
G3	40 (55.5%)

**Table 2:** AMH concentration in relation to FIGO stage.

	FIGO stage	Statistical characteristics							Mann-Whitney *U* test
		Mean	SD	Min	Q1	Q2	Q3	Max	
AMH/MIS (ng/mL)	I/II	0.39	0.65	0.00	0.00	0.1	0.34	1.75	0.702
	III/IV	0.34	0.79	0.00	0.00	0.08	0.33	3.26	

AMH/MIS: anti-Müllerian hormone/Müllerian inhibiting substance; FIGO: Federation Internationale de Gynecologie et d'Obstetrique; Max: maximum value; Min: minimum value; Q1: lower quartile; Q2: median; Q3: upper quartile; SD: standard deviation.

**Table 3:** AMH concentration in relation to grading.

	Grading	Statistical characteristics							*P* value Kruskal-Wallis test
		Mean	SD	Min	Q1	Q2	Q3	Max	
AMH/MIS (ng/mL)	G1	0.17	0.21	0.00	0.00	0.1	0.34	0.58	0.875
	G2	0.37	0.75	0.00	0.00	0.04	0.37	3.26	
	G3	0.3	0.62	0.00	0.00	0.01	0.31	2.95	

AMH/MIS: anti-Müllerian hormone/Müllerian inhibiting substance; Max: maximum value; Min: minimum value; Q1: lower quartile; Q2: median; Q3: upper quartile; SD: standard deviation.

**Table 4:** AMH concentration in relation to histological type of cancer.

	Histological type	Statistical characteristics							*P* value Mann-Whitney *U* test
		Mean	SD	Min	Q1	Q2	Q3	Max	
AMH/MIS (ng/mL)	Serosum	0.23	0.49	0.00	0.00	0.04	0.3	3.26	0.653
	Others	0.68	1.04	0.00	0.00	0.02	1.44	2.95	

AMH/MIS: anti-Müllerian hormone/Müllerian inhibiting substance; Max: maximum value; Min: minimum value; Q1: lower quartile; Q2: median; Q3: upper quartile; SD: standard deviation.

**Table 5:** Prognostic factors for overall survival selected by Cox's univariate analysis.

	Statistical characteristics					
	Parameter evaluation	Chi-squared	*P* value	HR	95% CI HR lower endpoint	95% CI HR upper endpoint
Age	0,02	3,66	0,06	1,03	0,99	1,05
AMH (ng/mL)	−0,01	0,66	0,41	0,98	0,95	1,02
Histo-Pat (serosum)	0,05	0,07	0,79	1,1	0,53	2,27
Grading (G2 + G3)	−1,04	4,24	0,04	0,12	0,02	0,9
FIGO (III/IV)	−0,83	10,04	0,001	0,19	0,06	0,53

AMH/MIS: anti-Müllerian hormone/Müllerian inhibiting substance; CI: confidence interval; FIGO: Federation Internationale de Gynecologie et d'Obstetrique; HR: hazard ratio.

**Table 6:** Prognostic factors for overall survival selected by Cox's multivariate analysis.

	Statistical characteristics				
	Parameter evaluation	*P* value	HR	95% CI HR lower endpoint	95% CI HR upper endpoint
Age	0,02	0,38	1,02	0,98	1,06
AMH (ng/mL)	−0,02	0,22	0,97	0,94	1,01
Histo-Pat (serosum)	0,08	0,7	1,17	0,51	2,67
Grading (G2 + G3)	−0,67	0,2	0,26	0,03	2,01
FIGO (III/IV)	−0,73	0,01	0,23	0,08	0,68

AMH/MIS: anti-Müllerian hormone/Müllerian inhibiting substance; CI: confidence interval; FIGO: Federation Internationale de Gynecologie et d'Obstetrique; HR: hazard ratio.
